# Connective-Tissue Growth Factor (CTGF/CCN2) Induces Astrogenesis and Fibronectin Expression of Embryonic Neural Cells *In Vitro*

**DOI:** 10.1371/journal.pone.0133689

**Published:** 2015-08-04

**Authors:** Fabio A. Mendes, Juliana M. Coelho Aguiar, Suzana A. Kahn, Alice H. Reis, Luiz Gustavo Dubois, Luciana Ferreira Romão, Lais S. S. Ferreira, Hervé Chneiweiss, Vivaldo Moura Neto, José G. Abreu

**Affiliations:** 1 Instituto de Ciências Biomédicas, Programa de Biologia Celular e do Desenvolvimento, Universidade Federal do Rio de Janeiro, Rio de Janeiro, RJ, Brazil; 2 Inserm, UMR894, Team Glial Plasticity, University Paris Descartes, Paris, France; 3 Instituto Estadual do Cérebro Paulo Niemeyer (IEC), Rio de Janeiro, RJ, Brazil; 4 Universidade Federal do Rio de Janeiro, pólo Xerém, Rio de Janeiro, RJ, Brazil; University of São Paulo, BRAZIL

## Abstract

Connective-tissue growth factor (CTGF) is a modular secreted protein implicated in multiple cellular events such as chondrogenesis, skeletogenesis, angiogenesis and wound healing. CTGF contains four different structural modules. This modular organization is characteristic of members of the CCN family. The acronym was derived from the first three members discovered, cysteine-rich 61 (CYR61), CTGF and nephroblastoma overexpressed (NOV). CTGF is implicated as a mediator of important cell processes such as adhesion, migration, proliferation and differentiation. Extensive data have shown that CTGF interacts particularly with the TGFβ, WNT and MAPK signaling pathways. The capacity of CTGF to interact with different growth factors lends it an important role during early and late development, especially in the anterior region of the embryo. ctgf knockout mice have several cranio-facial defects, and the skeletal system is also greatly affected due to an impairment of the vascular-system development during chondrogenesis. This study, for the first time, indicated that CTGF is a potent inductor of gliogenesis during development. Our results showed that *in vitro* addition of recombinant CTGF protein to an embryonic mouse neural precursor cell culture increased the number of GFAP- and GFAP/Nestin-positive cells. Surprisingly, CTGF also increased the number of Sox2-positive cells. Moreover, this induction seemed not to involve cell proliferation. In addition, exogenous CTGF activated p44/42 but not p38 or JNK MAPK signaling, and increased the expression and deposition of the fibronectin extracellular matrix protein. Finally, CTGF was also able to induce GFAP as well as Nestin expression in a human malignant glioma stem cell line, suggesting a possible role in the differentiation process of gliomas. These results implicate ctgf as a key gene for astrogenesis during development, and suggest that its mechanism may involve activation of p44/42 MAPK signaling. Additionally, CTGF-induced differentiation of glioblastoma stem cells into a less-tumorigenic state could increase the chances of successful intervention, since differentiated cells are more vulnerable to cancer treatments.

## Introduction

The connective-tissue growth factor (CTGF/CCN2) was first described in 1991 by Bradham and coworkers, as a novel growth factor secreted by human umbilical-vein endothelial cells (HUVECs), with high chemotactic and mitogenic activity [1–2]. Since then, an increasing amount of data has revealed several functions of CTGF, which seem to be related to its modular architecture [3–10].

The amino-acid sequence of CTGF is characterized by the presence of an N-terminal secretory signal and four distinct domains: a domain that shows homology to the IGF-binding protein, a von Willebrand factor type C repeat (VWC) or chordin-like cysteine-rich (CR) domain, a thrombospondin type 1 repeat (TSP-1) and a cysteine knot in the carboxy-terminal domain. This modular architecture is also found in NOV, CYR61, WISP1 and WISP3, which together with CTGF form the so-called CCN family of secreted factors [11].

The characteristic modular architecture of CTGF is responsible for the modulation of growth-factor signaling by binding and sequestering different ligands. Abreu et al. (2002) demonstrated that CTGF directly binds BMP4 and TGFβ-1 through its CR domain. CTGF can antagonize BMP4 signaling by blocking its binding to receptors, and has an opposite effect on TGFβ-1, enhancing its binding to receptors. CTGF cooperates with TGFβ-1 in most TGFβ responses, such as induction of the expression of extracellular matrix components, fibroblast proliferation, wound repair and fibrotic disorders [12–16]. A SMAD binding element was found within the ctgf promoter, indicating that ctgf is a direct target of TGFβs [14]. Indeed, fibroblasts produce high levels of CTGF mRNA and protein after activation with TGFβ-1 [3].

Mercurio et al. (2004) demonstrated that CTGF also modulates both canonical and non-canonical WNT signaling. CTGF interacts with the WNT pathway through a domain distinct from the TGFβ/BMP interacting domain. In this case, the CT domain of CTGF is important in the interaction with all four EGF repeats of the WNT co-receptor LRP6, and can also bind weakly to the WNT receptor Frizzled. CTGF competes with WNT for binding to LRP6 and Frizzled, blocking the access of WNT to its receptor. Moreover, CTGF was able to inhibit convergent extension movements of *Xenopus laevis* animal caps, also indicating its ability to control the non-canonical WNT pathway; however, the mechanism is not completely understood since the non-canonical pathways comprise several β-catenin-independent cascades [7]. In addition, CTGF may also be a downstream target of WNT/β-catenin signaling. Luo et al. (2004) showed that CTGF is upregulated at the early stages of BMP-9 and WNT-3a stimulation of osteoblast differentiation, and that CTGF expression is β-catenin-dependent [17].

CTGF expression can also be controlled by mitogen-activated protein (MAP) kinase pathways [18–20]. The three major MAP kinase pathways, extracellular signal-regulated kinases (ERK, also known as p42/44 MAPK), p38 and jun N-terminal kinase (JNK) serve as mediators for a variety of signaling molecules and growth factors [21–23]. ERK is classically activated via the sequential activation of Ras G proteins, Raf kinases and MEK1 and 2. MEKs, in turn, phosphorylate and activate ERK1 and 2. The minimal consensus phosphorylation motif in MAPK substrates is [Ser/Thr]-Pro. Activated MAPKs can phosphorylate a large number of intracellular substrates, leading to diverse cellular outcomes. ERK activates CTGF expression in different cell types including fibroblasts, mesangial cells and osteoblast cells [18–20, 24].

During the development of the nervous system, all cell types in the cortex are generated following a distinct temporal program. Neurons are differentiated with a peak at embryonic day 14 (E14); the astrocytes appear second, with a peak at postnatal day 2 (P2); and then the oligodendrocytes with a peak at P14. Since both neurons and astrocytes are derived from the same progenitor, a neurogenic to gliogenic switch has to occur during the temporal differentiation program. BMPs and Notch are both important signals involved in astrogenesis. BMP-2, together with gliogenic cytokines, causes the formation of a SMAD/STAT complex that activates transcription of gliogenic genes. Notch signaling can also promote astrogenesis in the presence of an active JAK-STAT signal. The ERK pathway also has an important role during the neural-cell differentiation program. During early stages, ERK pathway directly represses gp130 and the JAK-STAT pathway, promoting neurogenesis. However, some data have pointed to a pro-gliogenic role for the ERK pathway. Rajan and McKay (1998) showed that CNTF activates the JAK-STAT and MAPK pathways in multipotent stem cells of the central nervous system, to instruct them to an astrocytic fate. Moreover, Lee et al. (2004) showed that the cytokine erythropoietin is able to promote astrogenesis, activating the ERK pathway, and Nakanishi et al. (2007) showed that IL-6 and LIF also activate the ERK pathway, together with JAK-STAT during astrogenesis [25].

Considering that several signaling pathways modulated by CTGF are important in nervous-system development, this study was undertaken to determine whether CTGF can be important for the differentiation program of central nervous system cells. In order to achieve this, we added recombinant CTGF protein [5] to a culture of mouse embryonic E14 hemisphere cells. Our results showed that the exogenous CTGF protein increased the number of glial fibrillary acidic protein (GFAP) and GFAP/Nestin-positive cells without changing the proliferation index. Surprisingly, CTGF was also able to increase the number of Sox2-positive cells. Moreover, the exogenous CTGF protein was able to induce phosphorylation of ERK proteins and also increased the expression and deposition of the extracellular matrix protein fibronectin. Finally, using human malignant glioma stem cells (OB1 cell line), we showed that CTGF treatment induced GFAP, CD133, Nestin and inhibited Sox2 expression, suggesting a possible role for CTGF not only during normal brain development but also in the glioma differentiation program.

## Methods

### Primary embryonic neural cell culture

Mice experiments were carried out according to the guidelines granted by the Animal Care and Use Ethic Committee (Comissão de Ética no Uso de Animais—CEUA) of the Federal University of Rio de Janeiro and were approved by this committee under the permission number DHE-ICB 015. The experiments were also approved by the Brazilian Ministry of Health Ethics Committee (CONEP No. 2340). The mice were euthanized in CO_2_ chamber followed by cervical dislocation. The primary cultures were established as previously described [26–28]. Briefly, 14-day-old mouse embryo cerebral hemispheres were dissected in phosphate-buffered saline (PBS), stripped of meninges, minced and mechanically dissociated. Aggregates were removed by decantation and cells were obtained by centrifugation at 180 x g for 4 min. The pellet was resuspended in DMEM-F12 (Invitrogen) enriched with 2 nM glutamine and 14 mM sodium bicarbonate with 10% FBS (Invitrogen) and plated at a density of 5 x 10^6^ cells in 100-mm cell culture dishes or of 2 x 10^5^ cells in 24-well plates, with glass coverslips previously treated with poly-L-ornithine (1.5 μg/mL, PM 41000, Sigma). These cultures were kept in 37°C at 5%/95% CO_2_/H_2_O atmosphere for 5 days. The medium was changed 24 and 72 h after plating and on the last day, 30 min before processing for immunocytochemistry or total protein extraction. In all the experiments, 5nM CTGF was re-added in each medium change. The recombinant protein CTGF-Flag was obtained as described [5]. *Xenopus laevis* full-length Flag-CTGF from S2 cells was affinity purified with anti-Flag M2 affinity gel column and eluted with Flag peptide according to manufacturer’s directions (Sigma, St Louis, MO).

### Human glioblastoma stem cell line

The cell line of non-adherent human glioblastoma stem cells (OB1) were established as described in Patru et al., 2010. OB1 were grown in 75 cm^2^ tissue culture flasks (2500–5000 cells/ cm^2^) in DMEM-F12, supplemented with B27, N2 and G5 (Invitrogen, France). All cultures were maintained at 37°C in humidified atmosphere with 5% CO_2_.

### Immunocytochemistry

The cells were fixed with 4% paraformaldehyde for 15 min at room temperature. Fixed cultures were washed with PBS, permeabilized with 0.2% Triton X-100 in PBS for 3 min, and blocked for 1 h with 5% bovine serum albumin (BSA). Next, the cells were incubated with anti-BrdU (Sigma, 1:500), polyclonal anti-fibronectin (Sigma, 1:400) and anti-laminin (Sigma, 1:50), anti-Sox2 (Millipore, 1:100), anti-CD133 (Millipore, 1:20) antibodies or double-stained with polyclonal anti-GFAP (DAKP, 1:500) and monoclonal anti-Nestin (BD Biosciences 1:50) antibodies overnight at 4°C. Incubation with specific secondary Alexa Fluor 546 goat anti-mouse IgG (Molecular Probes 1:800), Alexa Fluor 488 goat anti-rabbit (Molecular Probes 1:400) or Alexa Fluor 546 goat anti-rat IgG (Molecular Probes 1:500) proceeded for 1 h at room temperature. Then, the cells were washed with PBS, stained with DAPI (Sigma), washed again and mounted. For negative controls, cells received similar treatment but the primary antibodies were omitted. The slides were observed in a Nikon TE 2000 inverted microscope. Images were captured using a CoolSNAP-Pro (Media Cybernetics) digital camera.

### BrdU incorporation assay

5’-Bromo-2’-deoxyuridine (BrdU, Sigma) was administered in the culture, with or without CTGF, for de last 2 h of treatment, at a final concentration of 10 μM. After fixation with 4% paraformaldehyde and permeabilization with 0.4% Triton X-100, cell were treated with 2 N HCl, then neutralized with 0.1 M Na_2_B_4_O_7_. After PBS washes, the rat anti-BrdU antibody (1:500) was incubated. The Cy3-labeled goat anti-rat antibody was used as a secondary antibody. A negative control for the BrdU assay was performed by omitting the primary antibody during immunostaining.

### [^3^H]-Thymidine incorporation assay

The cultured cells were seeded at 5x10^4^ cells per well in DMEM-F12 with 10% FBS in 24-well plates. In the last 6 hours of CTGF treatment, a pulse of [^3^H]-thymidine was added. After the five days of CTGF treatment, the medium was carefully removed and 300 μL of ice-cold 10% thrichloroacetic acid was added. Cells were harvested and [^3^H]-thymidine incorporation was measured on a scintillation counter.

### Cell viability assay

To measure the viability of the cells in culture we used the premixed WST-1 reagent (Clontech). This colorimetric assay is based on the cleavage of the tetrazolium salt WST-1 to a formazan-class dye by mitochondrial succinate-tetrazolium reductase in viable cells. 5x10^4^ cells were plated in a 96-well plate previously treated with poly-L ornithine (1,5μg/mL, PM 41000, Sigma) and cultured in presence of recombinant CTGF protein for 24, 48, 72, 96 e 120 hours. After each 24 hours, the reagent was added according to manufactures protocol and the absorbance was read at 450nm.

### Immunoblotting

The cultures were lysed in 50 μl of RIPA buffer (0.05 M Tris-HCl pH 7.4, 0.15 M NaCl, 1% NP-40, 2 mM EDTA, 1 mg/mL pepstatin, 1 mM PMSF, 1 mM NaF, 1 mM Na_3_VO_4_) 30 min after the last medium change. Protein concentration was measured by the Bradford method. Before loading, samples were mixed with sample buffer (200 mM DTT 4% SDS, 125 mM Tris pH 6.8, 20% glycerol and 0.02% bromophenol blue). Samples were heated for 5 min at 95°C and the proteins were separated in SDS-PAGE. Proteins were transferred to PVDF membranes (Amersham Biosciences) in transfer buffer (25 mM Tris, 192 mM glycine, 0.1% SDS and 20% methanol). Membranes were blocked in 5% BSA for phosphorylated proteins or in nonfat dry milk for all others, in TBSt (20 mM Tris pH 7.6, 137 mM NaCl and 0.1% Tween 20). Primary polyclonal antibodies anti-total p44/p42 MAPK (Cell Signaling 1:1000), anti-phosphorylated p44/p42 MAPK (Cell Signaling 1:1000), anti-phosphorylated p38 (Cell Signaling 1:1000), anti-total SAPK/JNK (Cell Signaling 1:1000), anti-phosphorylated SAPK/JNK (Cell Signaling 1:1000), anti-fibronectin (Sigma 1:1000), anti-laminin (Sigma 1:400), anti-tubulin (Sigma 1:1000), monoclonal anti-tubulin (Sigma 1:2000), monoclonal anti-β-tubulin (Covance, Eurogentec, Belgium), polyclonal anti-GFAP (Dakocytomation, France), polyclonal anti-OLIG-2 (R&D systems, France) and monoclonal anti-Sox2 (Neuromics, Acris, Germany) in blocking solution were incubated with the membranes overnight at 4°C, followed by incubation with Peroxidase-conjugated anti-rabbit IgG and peroxidase-conjugated anti-mouse IgG secondary antibodies (Amersham Biosciences 1:2000). The immunoblot reaction was developed with Super Signal West Pico chemiluminescent substrate (Pierce, USA) and exposed in Kodak X-OMAT film.

### Densitometries and statistical analysis

Densitometric analyses of the immunoblots were performed using ImageQuant software. The ratio between phosphor-specific bands and their respective loading controls was calculated and the results were normalized to the respective control groups. Nonparametric statistical tests were performed (Student’s *t* test) with GraphPad PRISM software (GraphPad Software, Inc., San Diego, CA). The results were considered to be significant when *P* < 0.05.

## Results

### Recombinant CTGF protein increased the number of GFAP and Sox2-positive cells *in vitro*

In order to test the ability of CTGF to affect the differentiation program of neural progenitors during development, we dissociated cells from 14-day-old mouse embryo cerebral hemispheres and grew them in DMEM-F12 (Invitrogen) with 10% fetal bovine serum (FBS, Invitrogen) in the presence of 5 nM recombinant CTGF protein [5]. Cells were maintained in culture for 5 days and the medium was replaced 24 and 72 h after plating and 30 min before fixation. 5nM recombinant CTGF protein was added after each medium change. Control cultures were maintained in DMEM-F12 supplemented with 10% FBS. Then, cells were processed for double immunocytochemistry against GFAP and Nestin or against Sox2.

**Fig 1:**
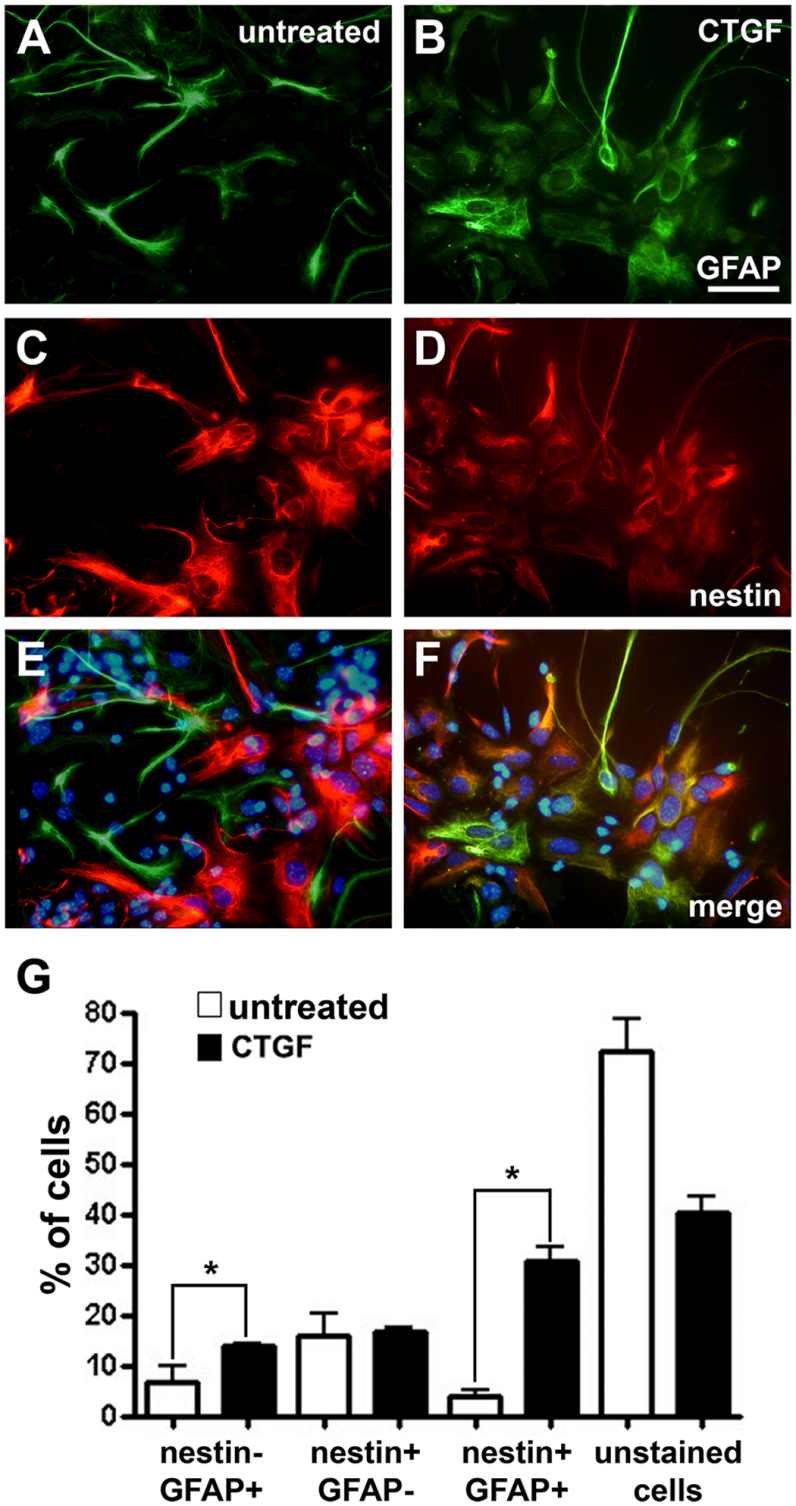
Exogenous CTGF protein increases the number of GFAP-positive cells of a neural progenitor culture. Double immunostaining showing GFAP (A and B green) and Nestin (C and D red) expression of untreated and CTGF-treated astrocytes. E and F show merged pictures of GFAP and Nestin immunostaining together with nuclei-DAPI staining. Scale bars 50 μm. G shows the percentage of cells that were unstained or stained with GFAP and/or Nestin. CTGF was able to increase the number of GFAP-positive cells and, more dramatically, the number of Nestin/GFAP-positive cells.

**Fig 2:**
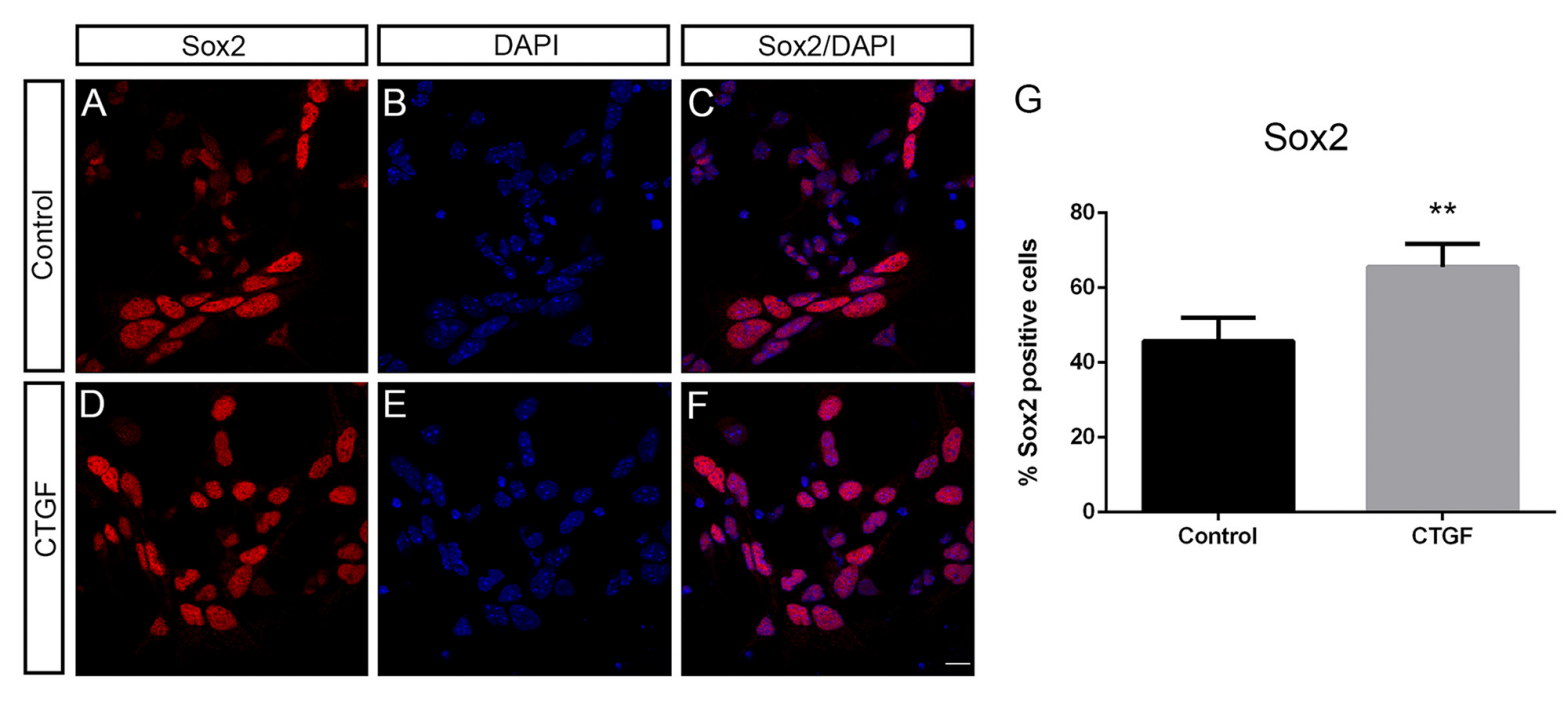
Exogenous CTGF protein increases the number of Sox2-positive cells of a neural progenitor culture. Immunostaining showing Sox2 (A and D) expression of untreated and CTGF-treated cells. C and F show merged pictures of Sox2 immunostaining together with nuclei-DAPI staining. Scale bars 10 μm. G shows the percentage of cells that were positive for Sox2.

After 5 days of culture, it was possible to identify cells that stained only for GFAP, some that stained only for Nestin, others that double-stained for GFAP/Nestin, and a large number of unstained cells (Fig 1). Interestingly, almost twice as many Nestin-/GFAP+ cells were observed when the cells were grown in the presence of CTGF (Fig 1A and 1B and Nestin-/GFAP+ bars in Fig 1G, *P* = 0.0215), and six times as many cells double-stained for Nestin and GFAP (Fig 1A–1F and Nestin+/GFAP+ bars in Fig 1G, *P* = 0.0103). However, no changes were observed in the number of cells that stained only for Nestin (Fig 1C–1F and Nestin+/GFAP- bars in Fig 1G). In addition, we observed a 43% decrease in the percentage of cells that did not stain for GFAP or Nestin (Fig 1E and 1F and Nestin-/GFAP- bars in Fig 1G, *P* = 0.0277). Finally, the percentages of beta-III tubulin-positive cells were the same in both CTGF-treated and untreated cultures (data not shown). In order to check the effect of CTGF treatment on the number of neural progenitors we also stained CTGF-treated and untreated cells for Sox2. Surprisingly, CTGF was able to increase 20% the number of Sox2-positive cells (Fig 2).

### Recombinant CTGF did not affect the proliferation rate of neural cells *in vitro*

Since it has been shown that CTGF induces proliferation in different cell types, we asked if addition of recombinant CTGF in neural progenitor-cell cultures derived from 14-day-old embryo hemisphere-cell cultures cultivated for 5 days could affect the cell growth rate. As described above, 5x10^4^ cells were plated in a 96-well plate and cultivated in the presence of 5 nM recombinant CTGF protein in DMEM-F12 with 10% FBS for 5 days. The medium containing CTGF was changed 24 and 72 h after plating and 30 min before the cells and controls were maintained in DMEM-F12 with 10% FBS. To test for changes in the proliferation index, we performed a WST-1 cell proliferation assay with these cells during the 5 days in culture. WST-1 reagent was added to the CTGF-treated cultures every 24 h during the 5 days of culture. The absorbance at 450 nm showed no differences in the proliferation index between untreated and CTGF-treated cells (S1 Fig). We also performed other proliferation assays on the 5^th^ day in culture, including incorporation of 5-bromo-2-deoxyuridine (BrdU) (S2 Fig) and incorporation of [^3^H] thymidine (S2 Fig). Both assays indicated no changes in the proliferation rate.

### Recombinant CTGF increased expression and deposition of fibronectin

**Fig 3:**
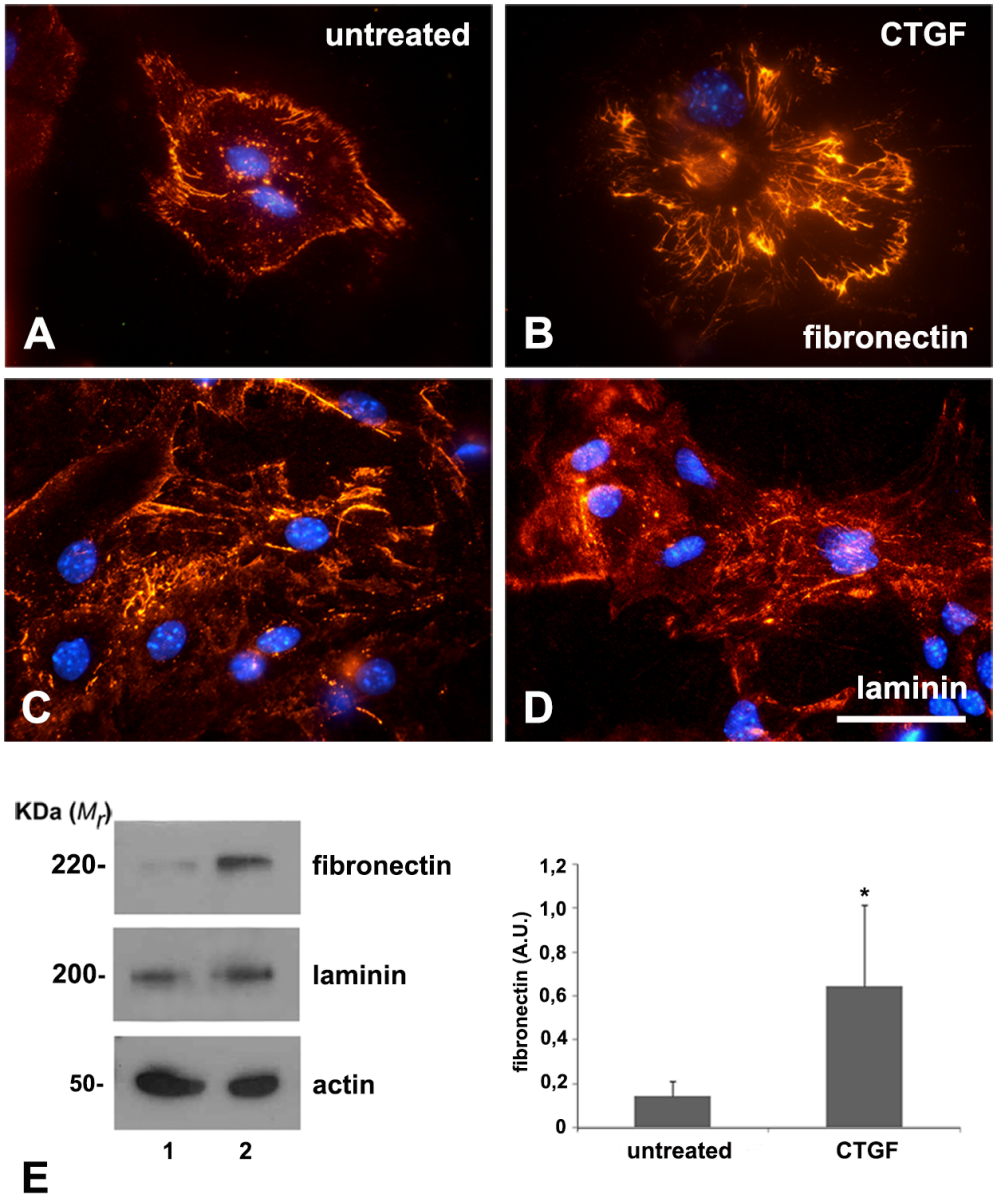
Recombinant CTGF increases expression and deposition of fibronectin. Immunostaining and immunoblotting for fibronectin and laminin. (A-D) Untreated or CTGF-treated progenitor neural cells immunostained for fibronectin (A, B) and laminin (C, D). (E) Immunoblotting for fibronectin and laminin of untreated (lane 1) or CTGF-treated neural progenitor cells (lane 2). CTGF incubation of neural progenitor cells for 120 h increased the expression of fibronectin threefold (compare untreated versus CTGF bars of graph in E). Scale bar 50 μm.

CTGF is also known to induce expression of the extracellular matrix protein fibronectin. In order to investigate if CTGF is able to increase the expression and deposition of fibronectin and laminin, two major extracellular matrix proteins essential for neural-cell development, we immunostained progenitor cell cultures derived from 14-day-old embryo hemispheres and cultivated for 5 days in the presence of 5 nM recombinant CTGF protein. The medium was replaced every 24 and 72 h after plating and 30 min before fixing and processing for immunocytochemistry against fibronectin and laminin. We also performed immunoblot assays (as described in Methods) using total protein extracts of these neural-cell cultures after 5 days of the same treatments. Tubulin was used as a loading control. The CTGF increased the deposition of fibronectin in the extracellular matrix (Fig 3A and 3B) and increased the expression almost six fold (Fig 3E, *P* = 0.0171). Differently from the untreated condition, fibronectin showed a fibrillary organization after CTGF treatment (Fig 3A and 3B). However, CTGF seemed not to modify the expression or deposition of laminin (Fig 3C, 3D and 3E). Treated or untreated cells had the same organization of laminin in their extracellular matrix.

### CTGF increased phosphorylation of p44/42 MAPK (ERK1/2) but not p38 MAPK or p54/46 MAPK (SAPK/JNK)

**Fig 4:**
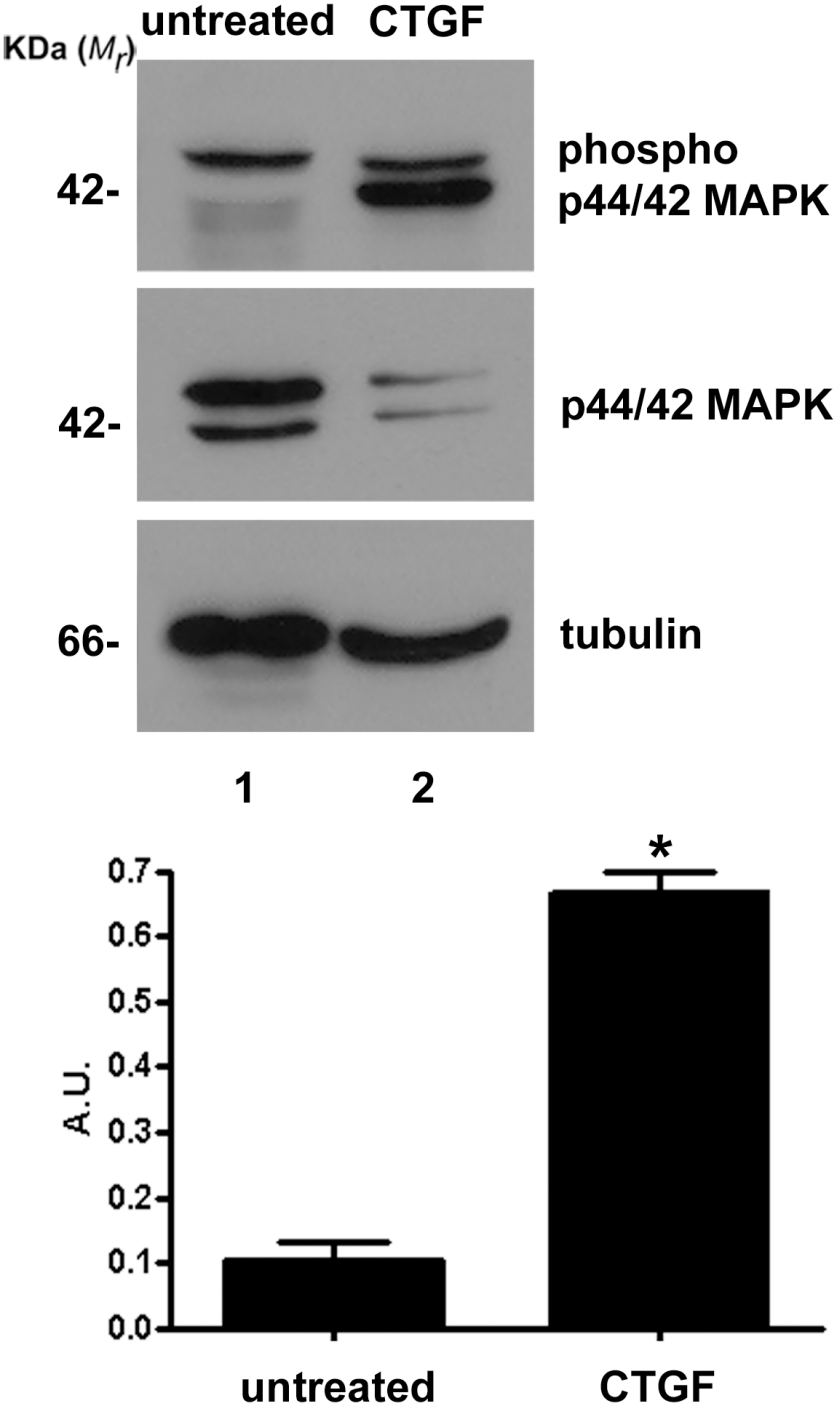
CTGF increases activation of p44/42 MAPK (ERK1/2). Immunoblot for phosphorylated p44/42 MAPK (ERK 1 and 2) of untreated or CTGF-treated neural progenitor cells. CTGF increased the expression of phosphorylated p44/42 MAPK almost sevenfold (compare lanes 1 and 2 and the graph of the densitometry). As a loading control we used total p44/42 MAPK and tubulin.

**Fig 5:**
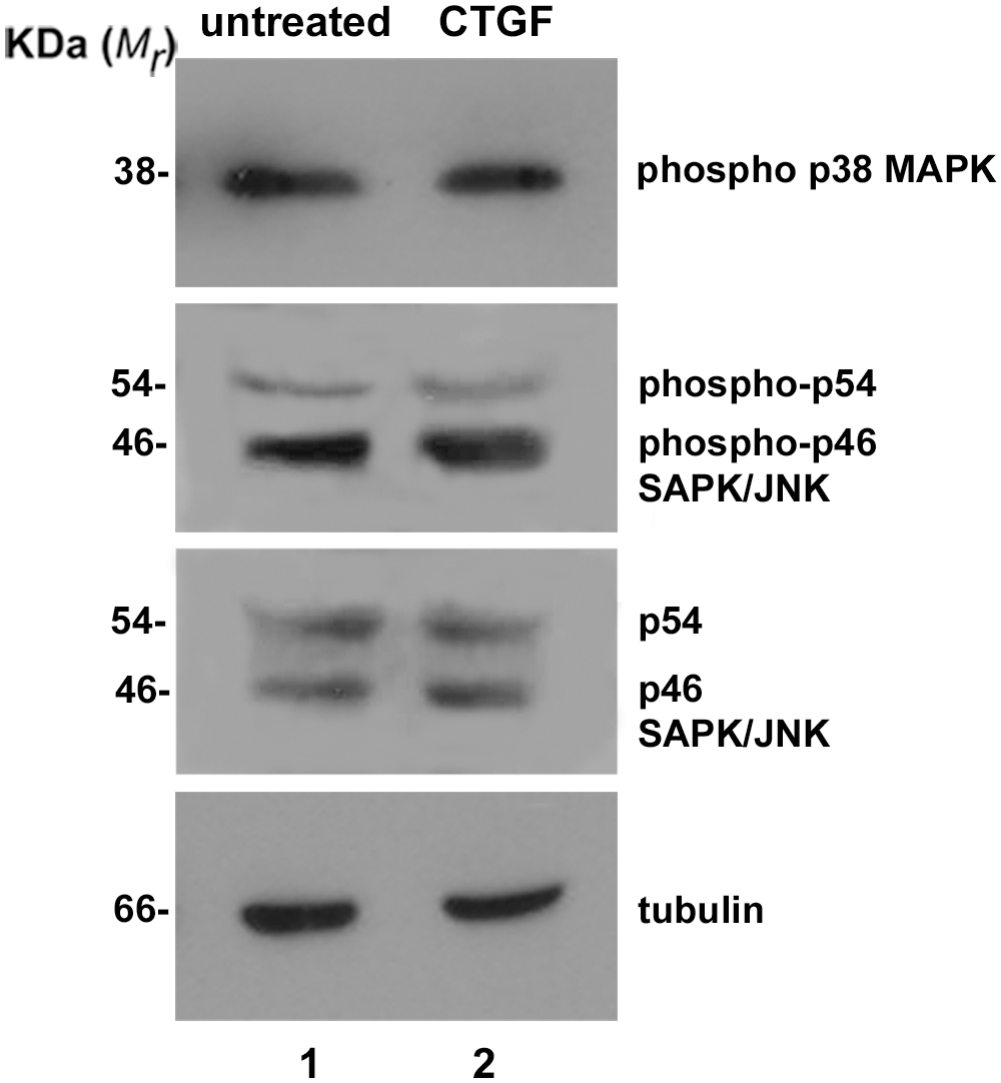
CTGF does not increase p38 MAPK or p54/46 SAPK/JNK MAPK pathways. Immunoblot for phosphorylated p38 MAPK and phosphorylated p46/p54 MAPK of untreated or CTGF-treated neural progenitor cells. CTGF did not change the levels of expression of these proteins (compare lanes 1 and 2). As a loading control we used total p46/p54 MAPK and tubulin.

Because p44/42 MAPK (ERK1/2) signaling is one of the principal pathways activated by CTGF and given that it has been shown to be involved in p44/42 MAPK (ERK1/2) activation and GFAP expression, we decided to test whether CTGF can modulate MAPK signaling effectors, phospho44/42 (ERK1/2), p38 and p54/46 (SAPK/JNK). Cell extracts from neural progenitor-cell cultures derived from 14-day-old mouse embryo hemispheres were separated on SDS-PAGE gels, and the proteins were transferred to PVDF membranes and probed with anti-phospho p44/42 MAPK, anti-phospho p38 MAPK and anti-phospho p54/46 SAPK/JNK antibodies. Tubulin was used as a loading control. As observed by densitometry, we demonstrated that addition of 5 nM recombinant CTGF protein in these cultures for 5 days dramatically increased (almost 7-fold) the phosphorylation of p44/42 MAPK (ERK1/2) level (Fig 4, compare lanes 1 and 2). This difference was highly significant by the Mann-Whitney test (*P* = 0.0181). In contrast, the same treatment did not produce any significant change in p38 MAPK or p54/46 SAPK/JNK phosphorylation (Fig 5, compare lanes 1 and 2).

### Recombinant CTGF increased GFAP and reduced Sox2 expression in human cancer stem cells

**Fig 6:**
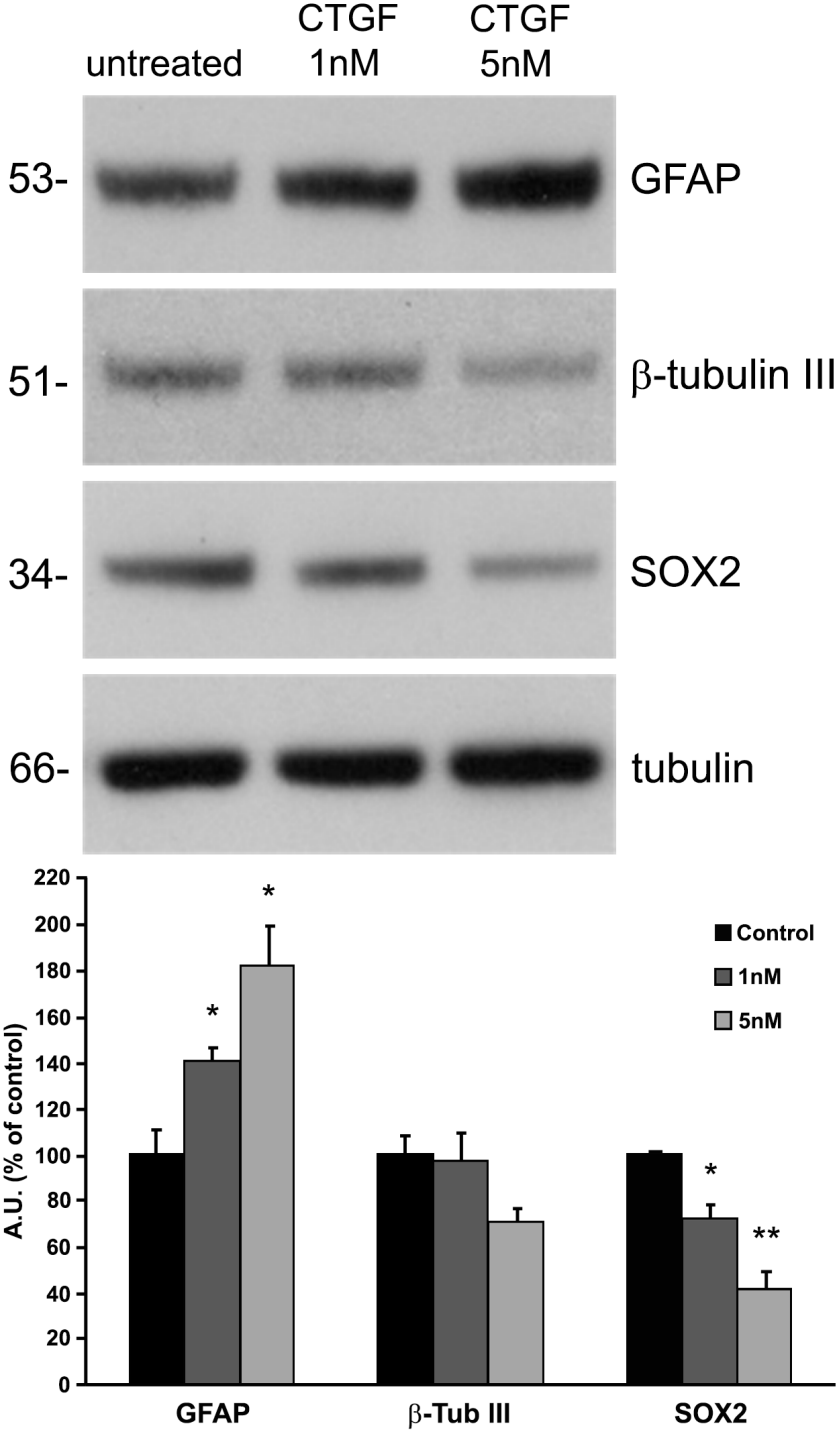
Exogenous CTGF protein increases GFAP and reduces Sox2 expression in human cancer stem cells. Immunoblotting showing GFAP, β-tubulin III and Sox2 expression in untreated and 1 nM and 5 nM CTGF-treated cells. CTGF incubation of human glioma stem cells for 120 h increased GFAP and reduced Sox2 expression in a dose-dependent manner. Alpha-tubulin was used as a loading control. **P < 0.001 and *P < 0.005.

**Fig 7:**
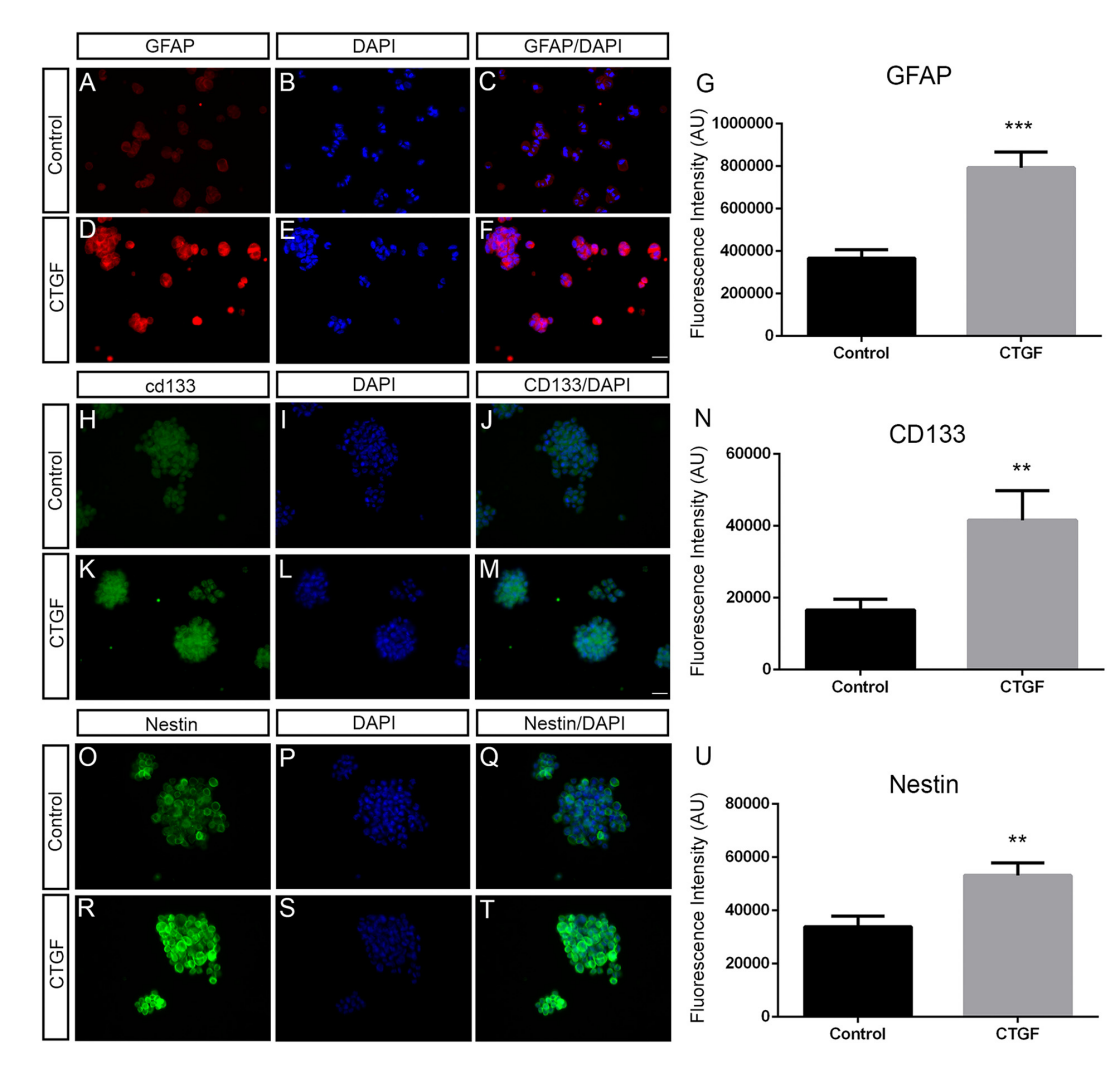
Exogenous CTGF protein increases the number of GFAP-, CD133- and Nestin-positive cells of human cancer stem cells. Immunostaining showing GFAP (A and D), CD133 (H and K) and Nestin (O and R) expression of untreated and CTGF-treated astrocytes. C and F, J and M and Q and T show merged pictures of GFAP, CD133 and Nestin immunostaining together with nuclei-DAPI staining respectively. Scale bars 25 μm. G, N and U show the fluorescence intensity of cells that were stained with GFAP, CD133 and Nestin respectively.

To investigate whether CTGF also plays an important role in neural cell differentiation in a human model, we treated OB1 cells [29], a malignant glioma stem cell line, with recombinant CTGF protein. Both 1 nM and 5 nM CTGF induced an astrocytic differentiation of these cells, as GFAP expression increased in a dose-dependent manner (Fig 6), 40% with 1 nM CTGF and 80% with 5 nM CTGF. Sox2 expression decreased in a dose-dependent manner (Fig 6), 30% with 1 nM CTGF and 60% with 5 nM CTGF. As expected, no significant difference was observed in β-tubulin III expression levels. These differences were highly significant by the Mann-Whitney test, *P* < 0.001 and *P* = 0.005 respectively. We also performed immunocytochemistry against the glial marker GFAP and the markers of neural progenitors, CD133 and Nestin. 5nM CTGF was able to increase approximately 2.0 fold the intensity of GFAP, 2.7 fold the intensity of CD133 and 1.6 fold the intensity of Nestin immunostaining (Fig 7).

## Discussion

In this study, we associated CTGF with astrocyte differentiation for the first time. Recombinant CTGF protein added during the first steps of the embryonic neural-cell differentiation process *in vitro* was able to increase the number of GFAP-positive cells and to activate p44/42 MAPK (ERK1/2). CTGF did not activate other MAPK pathways such as p38 or JNK. CTGF was also able to increase fibronectin expression and deposition in the extracellular matrix of these newly differentiated astrocytes. Together, these findings suggest that CTGF induces astrocytic differentiation and modulates the MAPK signaling pathway in the first steps of neural cells differentiation.

The CTGF protein contains different modules that capacitate CTGF to interact with a large number of cytokines, growth factors and different extracellular matrix proteins, modulating different signaling pathways [5, 7, 10, 30]. CTGF was first implicated in the promotion of cell adhesion and mitogenesis in both fibroblasts and endothelial cells and in the stimulation of cell migration in fibroblasts [1, 12, 31]. CTGF has also been extensively implicated in the differentiation of chondrocytes and osteoblasts [32–37] as well as in the differentiation of diverse cell types such as myofibroblasts, adipocytes, endothelial cells and mammary epithelial cells [4, 31, 38–40]. Very little is known about the roles of CTGF in neural development or neural-cell differentiation. CTGF is expressed in early stages of neural-tube development in both *Xenopus laevis* and the mouse. CTGF mRNA was detected in the floor plate and notochord of the *Xenopus* neural-plate stage [5] and its mRNA and protein staining was observed in the mouse neural tube as early as 10.5 days of gestation [34, 41]. Moreover, Freemantle et al. (2002)[42] showed by microarray that ctgf is one of 57 genes that are upregulated during retinoic acid-induced differentiation of the human embryonal carcinoma (EC) cell line, NT2/D1, toward a neuronal lineage. More recently, Obayashi et al. (2009) [43] also showed by microarray that ctgf is one of 45 genes upregulated during serum-induced differentiation of human neural progenitor cells into astrocytes. Our results showed that recombinant purified CTGF, added to the first steps of neural-cell differentiation *in vitro*, increased the number of cells that were immunostained for the astrocytic marker GFAP or cells double-marked for Nestin and GFAP, indicating an increase in the number of cells committed to the astrocytic phenotype. Although the maturity of these GFAP-positive cells was not revealed by these results, they suggest that these cells are in a transient stage, entering the astrocytic differentiation program. According to the literature, GFAP expression in primary cultures of radial glia starts after day 5, which correlates with the transition period of astrocyte maturation in rodents *in vivo* [44]. This result supports our *in vitro* system and indicates that CTGF’s role in the developing brain is related to the astrocyte differentiation program.

To induce cell differentiation, CTGF modulates important signaling pathways. Since most CTGF partners such as TGFβ-1, BMP, EGF and WNT are implicated in nervous-system development [45–47], we could predict an important role for CTGF in the differentiation process of neural cells during development. The main interaction of CTGF is with the TGFβ-1 signaling pathway. TGFβ-1 induces expression and secretion of CTGF, and CTGF binds directly to the TGFβ-1 ligand and increases its binding to the receptors, enhancing TGFβ-1/SMAD2 signaling as a positive feedback loop [5]. This partnership has been implicated in many diseases, including most types of fibrosis [48–51]. TGFβ-1/SMAD2 signaling also has a role during astrocyte fate commitment of embryonic neural-cell cultures [52–53]. TGFβ-1 protein activated SMAD2 translocation to the nucleus of early-differentiated astrocytes and increased the overall number of GFAP-positive cells in these cultures while it decreased the expression of progenitor markers, suggesting that TGFβ-1 drives progenitor cells toward an astrocyte phenotype [52]. It is still not known if CTGF can increase TGFβ-1 signaling in these astrocyte precursors or in any of the developing neural cells. However, a few studies suggest that this cooperation between CTGF and TGFβ-1 can occur during development [54–55]. In a previous study, our group showed that CTGF and components of the TGFβ-1 signaling pathway are expressed in the same structures during tooth development. Yet, CTGF seems not to cooperate with TGFβ-1 signaling in this situation since ctgf knockout mice did not show any reduction in the levels of phosphorylated SMAD2, suggesting that they might function independently [56].

CTGF also interacts with the MAPK signaling pathway. CTGF expression induced by TGFβ requires both phosphorylation of SMAD and p44/42 MAPK/ERK activation in rat mesangial cell line and human skin fibroblast primary cultures [18, 57]. The ERK pathway is required for CTGF to potentiate myofibroblast differentiation induced by TGFβ [58]. During chondrocyte differentiation, CTGF can block canonical signaling of BMP, a member of the TGFβ superfamily. However, it can also modulate non-canonical signaling pathways of BMP. CTGF inhibits BMP-induced ERK1/2 phosphorylation, blocking chondrocyte proliferation, and activates p38 MAPK signaling inducing chondrocyte differentiation [59, 37]. A vast amount of data implicates MAPK signaling pathways in astrocyte differentiation *in vitro* [53, 60–62]. Our results showed that CTGF increased more than six fold the phosphorylation of p44/p42 MAPK but not p38 MAPK or P54/p46 SAPK/JNK in astrocyte progenitor cells, suggesting that at least part of the CTGF function in astrocyte differentiation may involve activation of the specific MAPK signaling cascade. Another well-known function of CTGF is to coordinate extracellular matrix production and degradation during development and in pathological conditions [12, 63]. Nagai and coworkers [64] showed that CTGF activates both p44/42 and p38 MAPK in the human retinal pigment epithelial cell line, leading to the production of laminin and fibronectin. The induction of fibronectin expression by CTGF seemed to require p44/42 MAPK activation, while the induction of laminin seemed to require both p44/42 and p38MAPK activation [64]. Our results showed that CTGF is able to induce the expression and deposition of fibronectin but not of laminin in these newly differentiated astrocytes. This result is partly in agreement with the results of Nagai et al. (2009), in that CTGF is able to activate only p44/42 MAPK and not p38, which correlates with the induction of fibronectin but not laminin.

Laminin and fibronectin are the two main extracellular matrix proteins of the developing brain. They are important, for example, in cell adhesion, proliferation and axon migration during the development of the nervous system. The finding that CTGF induces the expression of fibronectin by astrocytes during development suggests a possible role for CTGF in cellular interactions and/or axon migration during the development of the nervous system. Our study provided the first evidence that CTGF induces astrocyte differentiation, activating the MAPK signaling pathway and promoting fibronectin expression and deposition.

Incubation of glioblastoma stem cells with CTGF protein increased the expression of GFAP and a decrease of Sox2 proteins, indicating an increase in the differentiation rate of these cells (Fig 6). Targeting glioblastoma stem cells may involve indirect and direct strategies. Indirect targeting strategies involve the perivascular [65], hypoxic [66] and immune [67] niches. Direct approaches involve overcoming the resistance of glioblastoma stem cells to standard treatment [68], blocking their functions [69], and inducing differentiation into a less-tumorigenic state that is more vulnerable to cancer treatments [70]. The precise mechanisms by which CTGF induces glioblastoma stem-cell differentiation, however, need to be investigated further. These data, together with previously published information, point to a more-complex mechanism that could involve multiple pathways. Therefore, it is likely that CTGF can interact with different pathways and promote a link between them, during the first steps of astrocyte and also during glioblastoma stem-cell differentiation.

## Supporting Information

S1 Fig Recombinant CTGF did not affect the proliferation rate of neural cells *in vitro*. (A) Graph showing the result of the WST-1 cell proliferation assay of untreated or CTGF-treated neural progenitor cells every 24 h after plating.
(TIF)

S2 Fig Recombinant CTGF did not affect the number of BrdU-positive cells *in vitro*. (A and B) Immunocytochemistry showing BrdU-positive cells of untreated or CTGF-treated neural progenitor cells after 120 h in culture. (C) Graph showing the percentage of BrdU-positive cells of untreated or CTGF-treated neural progenitor cells after 120 h in culture. (D) [^3^H] Timidin incorporation of untreated or CTGF-treated neural progenitor cells after 120 h in culture.
(TIF)
