# Supplementary figures and images for: Connective-Tissue Growth Factor (CTGF/CCN2) Induces Astrogenesis and Fibronectin Expression of Embryonic Neural Cells In Vitro
Source: PLoS One. 2015 Aug 4;10(8):e0133689. doi: 10.1371/journal.pone.0133689 (PMC4524627; doi:10.1371/journal.pone.0133689)

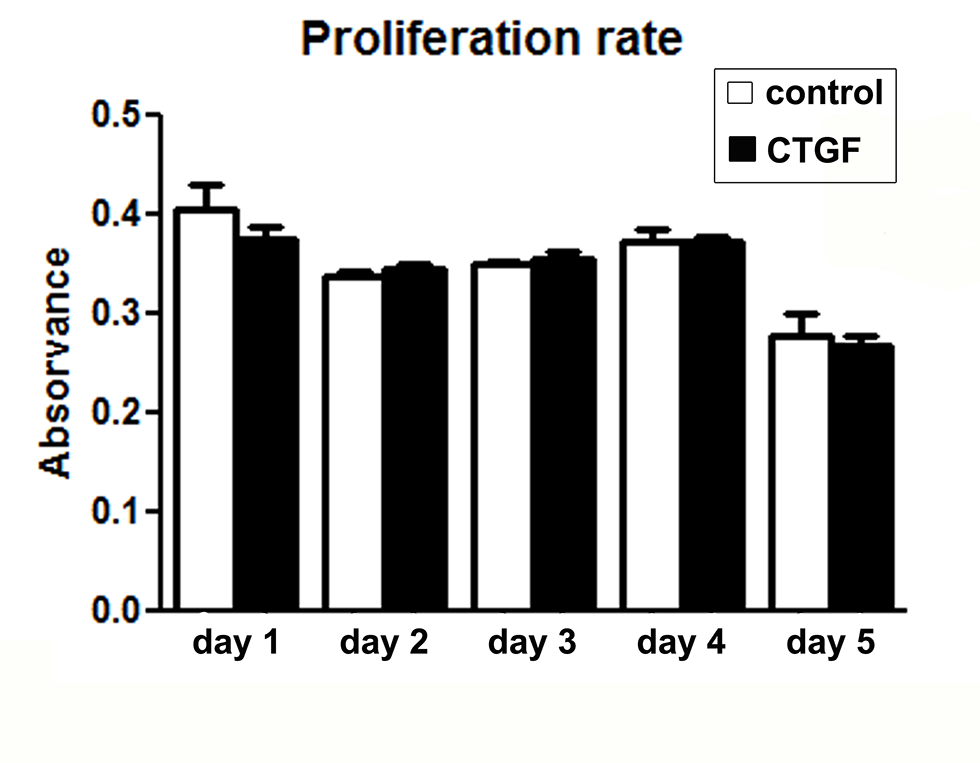

Supplement: S1 Fig — (A) Graph showing the result of the WST-1 cell proliferation assay of untreated or CTGF-treated neural progenitor cells every 24 h after plating. (TIF) [file pone.0133689.s001.tif]

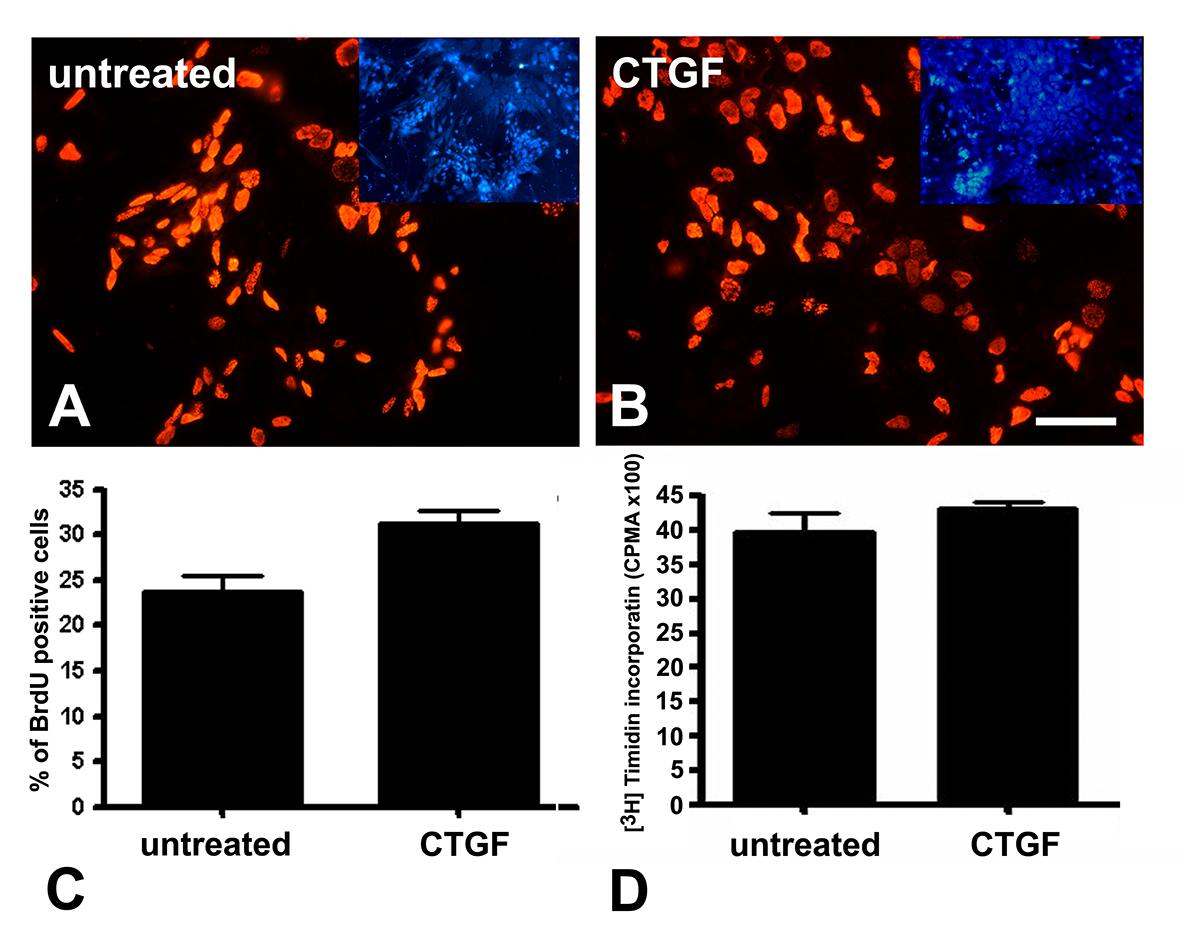

Supplement: S2 Fig — (A and B) Immunocytochemistry showing BrdU-positive cells of untreated or CTGF-treated neural progenitor cells after 120 h in culture. (C) Graph showing the percentage of BrdU-positive cells of untreated or CTGF-treated neural progenitor cells after 120 h in culture. (D) [3H] Timidin incorporation of untreated or CTGF-treated neural progenitor cells after 120 h in culture. (TIF) [file pone.0133689.s002.tif]
